# Genetic Evidence for a Putative B Cell–Cholate Immunometabolic Axis in Pericarditis: A Mendelian Randomization Study

**DOI:** 10.3390/biomedicines14092063

**Published:** 2026-09-14

**Authors:** Yifei Zhang, Wuxiao Yang, Xuewen Li

**Affiliations:** 1 Department of Cardiology, Third Hospital of Shanxi Medical University, Shanxi Bethune Hospital, Shanxi Academy of Medical Sciences, Tongji Shanxi Hospital, Taiyuan 030032, China; dr20250162273@sxmu.edu.cn; 2 Department of Cardiology, Shanxi Provincial People’s Hospital, Shanxi Medical University, Taiyuan 030012, China; 13753143192@163.com

**Keywords:** Mendelian randomization, immune cell phenotypes, pericarditis, cholate, genetic associations, plasma metabolites

## Abstract

**Background:** Pericarditis is an inflammatory heart disease with unclear causal mechanisms. Immune dysregulation is implicated, but observational studies cannot reliably infer causality. Whether plasma metabolites mediate immune-pericarditis pathways remains unknown. **Methods:** A two-sample Mendelian randomization (MR) study was performed using publicly available summary-level genome-wide association study data. Genetic instruments were selected via significance screening, linkage disequilibrium clumping, and strength evaluation (F-statistic > 10). A relaxed instrument selection threshold of *p* < 1 × 10^−5^ was used for immunophenotypes and metabolites, and a threshold of *p* < 5 × 10^−6^ was applied for pericarditis in reverse MR. Forward MR assessed potential causal effects of immune cells and metabolites on pericarditis; reverse MR tested the opposite direction. Two-step mediation MR evaluated whether metabolites mediated immune-to-pericarditis pathways. The primary method was inverse variance weighting (IVW), complemented by a range of sensitivity analyses. **Results:** A total of 18,621 SNPs served as instruments for immunophenotypes and 34,843 for metabolites. Forward MR identified 29 immunophenotypes with genetically predicted associations with pericarditis (20 risk, 9 protective). Reverse MR identified 13 immunophenotypes associated with genetic susceptibility to pericarditis, indicating a bidirectional genetic architecture. Ten plasma metabolites showed genetically predicted associations with pericarditis. After false discovery rate (FDR) correction, these key associations remained significant. Mediation analysis identified a putative immunometabolic pathway in which CD25 on IgD+ CD38br B cells increased pericarditis risk partly through downregulation of the protective metabolite cholate (β_1_ = −0.114, *p* = 0.002; β_2_ = −0.419, *p* = 0.001). The indirect effect was 0.048 (95% CI: 0.012 to 0.097), accounting for 32.8% of the total effect. All key results were robust in sensitivity analyses. **Conclusions:** This study provides genetic evidence supporting a putative immune cell–metabolite–pericarditis pathway involving CD25-positive B cells and cholate, and suggests potential immunometabolic targets for future investigation.

## 1. Introduction

Pericarditis is an inflammatory disease of the pericardium that imposes a substantial global health burden. Epidemiological studies estimate the annual incidence of pericarditis at 27 to 37 per 100,000 people, with mortality rates of approximately 1% to 2% among acute cases. Its etiology is heterogeneous and includes infections, autoimmune diseases, and post-myocardial infarction events, all of which can substantially affect quality of life [1]. Pericarditis can also lead to serious complications, including constrictive pericarditis and cardiac tamponade, which may require surgical intervention and increase morbidity [2]. More recently, immune-related adverse events, particularly those associated with cancer immunotherapies, have been recognized as an increasingly important cause of pericarditis, highlighting the need for a better understanding of its pathophysiology and risk factors [3]. Therefore, clarifying the causal relationships between immune responses, plasma metabolites, and pericarditis is important for the development of targeted therapeutic strategies [4,5].

Observational studies using conventional statistical methods are often plagued by basic problems such as reverse and confounding causation, where the causal relation between exposures and outcomes is not well understood. Although many studies have correlated immune cell profiles and pericarditis, it is inherently difficult to determine whether immune changes are a cause or a result of pericarditis [6,7]. Furthermore, the common use of self-reported data and cross-sectional study designs adds further biases that limit the validity of results [8]. These limitations require more powerful methodologies that are able to elucidate causal pathways and define unambiguous temporal relations between risk factors and disease outcomes. As a result, traditional approaches to pericarditis are not always suitable to unravel the complex connection between genetic predisposition, immune response and environmental factors.

Mendelian randomization (MR) is a robust methodological framework that utilizes genetic variants as instrumental variables (IVs) to deduce causality, effectively minimizing confounding biases inherent in conventional observational studies. Its logic is based on the random selection of alleles during gamete production, which mimics the structure of a randomized controlled trial, and thus is more robust in the evidence for causal inference [9]. This method has been successfully employed to describe causal relations between different exposures and health outcomes. Given the complex pathobiology between immunity and pericarditis and the incomplete understanding of its causal architecture, this study applied an MR framework to systematically assess the potential causal effects of immunophenotypes and plasma metabolites on pericarditis risk. To our knowledge, no previous study has integrated immune cell GWAS data, metabolomic GWAS resources, and mediation MR analysis in the context of pericarditis, thereby addressing an important evidence gap in this field [5,10]. Among plasma metabolites, bile acids have been increasingly implicated in immune regulation and cardiovascular biology, which supports the biological plausibility of the mediation analyses performed in this study. Accordingly, a two-sample MR analysis was conducted to explore the potential causal associations between plasma metabolites, immune cells, and pericarditis. This study will examine the central role of immune regulation in pericarditis pathogenesis and will provide genetic insight into the underlying metabolic mediation mechanisms with the eventual goal of informing novel approaches to the selective prevention and management of this condition.

## 2. Methods

### 2.1. Study Design

**Figure 1:**
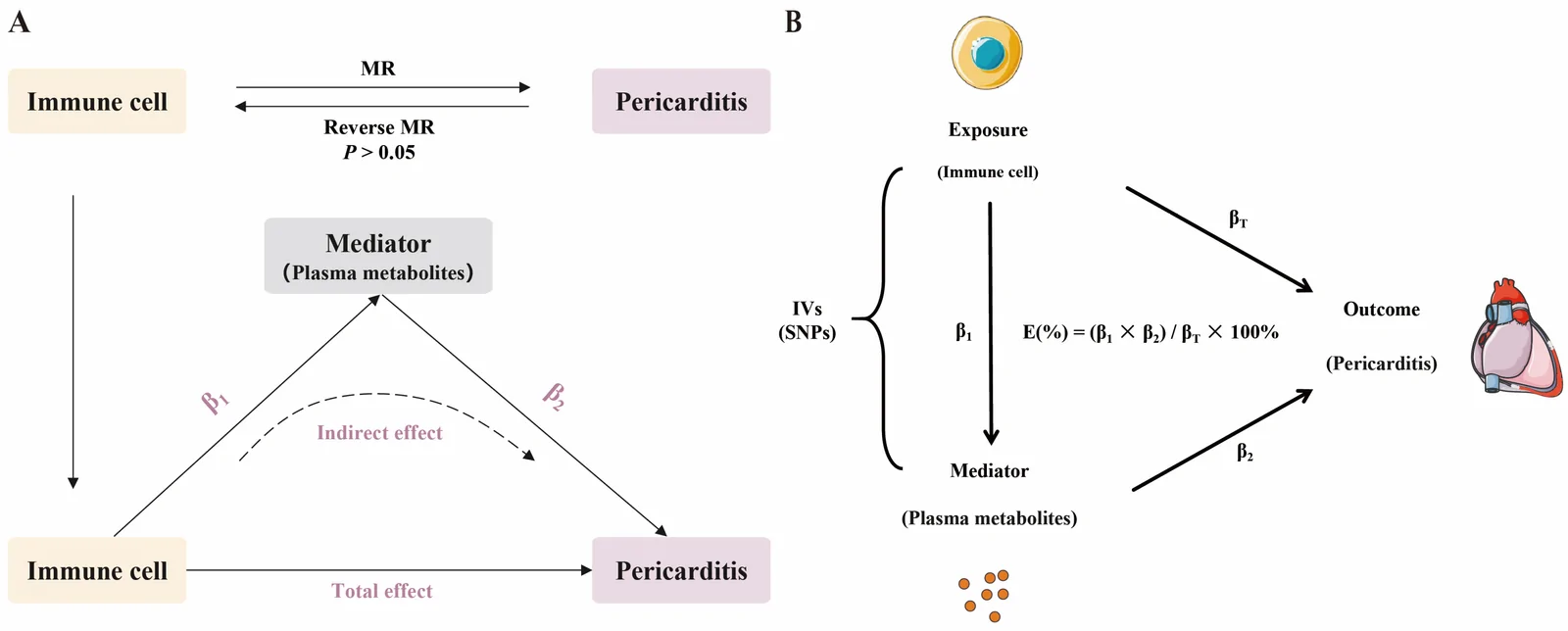
Study design overview and analytical framework for MR mediation analysis. (**A**) Flowchart of the research workflow, including data sources and the sequential analytical procedures: (1) forward MR to identify candidate causal immunophenotypes for pericarditis, (2) reverse MR to test for reverse causality, and (3) two-step MR to determine and quantify metabolite mediation. (**B**) Schematic of the two-step MR mediation model. Path coefficients β_1_ and β_2_ represent the exposure-mediator and mediator-outcome effects, respectively, estimated via MR. The total effect (β_T_) is estimated independently. The mediated effect is quantified as the product β_1_β_2_, and the proportion mediated is calculated as (β_1_β_2_)/β_T_. Arrows indicate the direction of the estimated effects among immune cells, plasma metabolites, and pericarditis: β_1_ (immune cells to plasma metabolites), β_2_ (plasma metabolites to pericarditis), and β_T_ (total effect from immune cells to pericarditis).

A two-sample MR design was used to systematically examine whether plasma metabolites mediate a potential causal pathway from immune cells to pericarditis (Figure 1). The reporting of this study followed the STROBE-MR guidelines (Supplementary Table S1) [11,12]. To accomplish this goal, three successive analyses were conducted. First, forward MR analyses were conducted to estimate the potential causal impact of 731 immunophenotypes (across 7 major categories, including B cells, cDCs, and TBNK cells) on pericarditis. Second, to assess potential reverse causality, bidirectional MR analyses were performed, in which pericarditis was treated as the exposure and the 731 immunophenotypes as outcomes; a further reverse MR analysis was also restricted to the immunophenotypes retained after forward screening. Finally, based on the findings from the first two steps, we used a two-step MR approach to test and quantify the mediation using the significant plasma metabolites identified as mediators to link immune cells to pericarditis. 

### 2.2. Data Sources

This study utilized publicly available summary-level data from “genome-wide association studies” (GWAS), all of which were based on European ancestry populations. Specifically, the genetic links for pericarditis were sourced from the GWAS Catalog (dataset ID: ebi-a-GCST90018896), comprising 455,165 participants (1795 cases and 453,370 controls) and covering 24,190,336 single-nucleotide polymorphisms (SNPs). Genetic instruments for 731 immunophenotypes were sourced from the GWAS Catalog (accession ID range: ebi-a-GCST90001391 to ebi-a-GCST90002121) as published by Orrù et al. [13]. Summary statistics for 1400 plasma metabolites were obtained from a recent GWAS (accession ID range: GCST90199621 to GCST90201020) by Chen et al. [14]. The exposure, mediator, and outcome GWAS datasets were derived from independent study populations of European ancestry, and no sample overlap was expected. Pericarditis status in the outcome GWAS was defined based on the source study; the dataset does not allow stratification by specific etiologies such as viral, autoimmune, or malignant pericarditis.

### 2.3. Selection of Instrumental Variables

To ensure that the IVs satisfied the core assumptions of relevance and independence [9], a multi-step selection procedure was implemented. First, to meet the relevance assumption [15], a relaxed instrument selection threshold was applied to ensure a sufficient number of genetic instruments for the subsequent mediation analyses. Specifically, SNPs associated with the exposure at *p* < 1 × 10^−5^ were selected as candidate IVs for the 731 immunophenotypes and 1400 plasma metabolites [16]. This threshold is intentionally less stringent than the conventional genome-wide significance threshold and was used as an exploratory instrument selection criterion rather than as a claim of genome-wide significance. For pericarditis as the exposure in reverse MR analyses, a more stringent threshold of *p* < 5 × 10^−6^ was applied to ensure adequate instrument strength for the smaller number of pericarditis cases. To guarantee instrument independence and reduce the bias caused by linkage disequilibrium (LD), we performed LD-based clumping based on a European reference panel (clumping window: 10,000 kb; LD threshold: R < 0.001) [17]. Finally, to reduce weak instrument bias, the F-statistic was calculated for each SNP and only SNPs with an F-statistic > 10 were included in the primary analyses [9]. For the reverse MR analyses, the retained instrument strength was assessed by the F-statistic; all SNPs used in the reverse analyses had F > 10, with a range of 20.87–26.93 for the key immunophenotype-specific analyses.

### 2.4. Statistical Analysis

Potential causal relationships were evaluated using five MR methods. Effect estimates for immune cell phenotypes are expressed per standard deviation increase in the respective phenotype. The inverse variance weighted (IVW) method was applied as the main analysis with the weighted median, MR-Egger, weighted mode, and simple mode methods for robustness testing [9,18,19]. In the discovery screening phase, associations were selected using nominal *p*-value thresholds combined with effect-direction consistency and sensitivity analyses, as described below. For forward screening of immune cell phenotypes, candidate associations were retained when the IVW estimate reached *p* < 0.05, the odds ratio (OR) direction was consistent across all five MR methods, and the MR-Egger intercept test showed no significant horizontal pleiotropy. For metabolite screening, a more stringent IVW threshold of *p* < 0.01 was applied, together with the same requirements for consistent effect direction and absence of significant pleiotropy. To further address multiple testing, the Benjamini–Hochberg false discovery rate (FDR) was applied as a sensitivity analysis for the main immune cell and metabolite associations; key findings remained significant after FDR correction. Moreover, to test the strength of the results, we performed a series of sensitivity analyses [20,21], including leave-one-out analysis to assess whether the findings were driven by any single influential SNP, MR-Egger intercept tests to evaluate horizontal pleiotropy, and Cochran’s Q tests to examine heterogeneity. MR-PRESSO was further applied to the key pathway analyses, including the total effect of CD25 on IgD+ CD38br on pericarditis and the association between cholate and pericarditis, to detect potential horizontal pleiotropy and identify outlier SNPs. Steiger directionality testing was also performed for the key immune cell–disease and metabolite–disease relationships, confirming that the instruments explained substantially more variance in the respective exposure than in the outcome. For these sensitivity analyses, a *p*-value > 0.05 was considered to indicate no strong evidence for horizontal pleiotropy or heterogeneity. Mediation analysis was conducted using a two-step MR to quantify the indirect effect of immune cells on pericarditis through metabolites [21]. This included estimating the following: (i) the total effect of the immunophenotype on pericarditis (β_T_); (ii) the effect of the immunophenotype on the mediator (β_1_); and (iii) the effect of the mediator on pericarditis (β_2_). The indirect effect was calculated as the product of the two path coefficients (β_1_ × β_2_). Its 95% confidence interval (CI) was estimated using the product-of-coefficients method with Monte Carlo simulations in RMediation and was further confirmed by the delta method. The direct effect was estimated as β_T_ − β_1_ × β_2_, and the proportion mediated was calculated as (β_1_ × β_2_)/β_T_. All analyses were done in R (version 4.5.1) and TwoSampleMR package (version 0.6.22).

## 3. Results

### 3.1. Genetic Associations Between Immune Cells and Pericarditis

**Figure 2:**
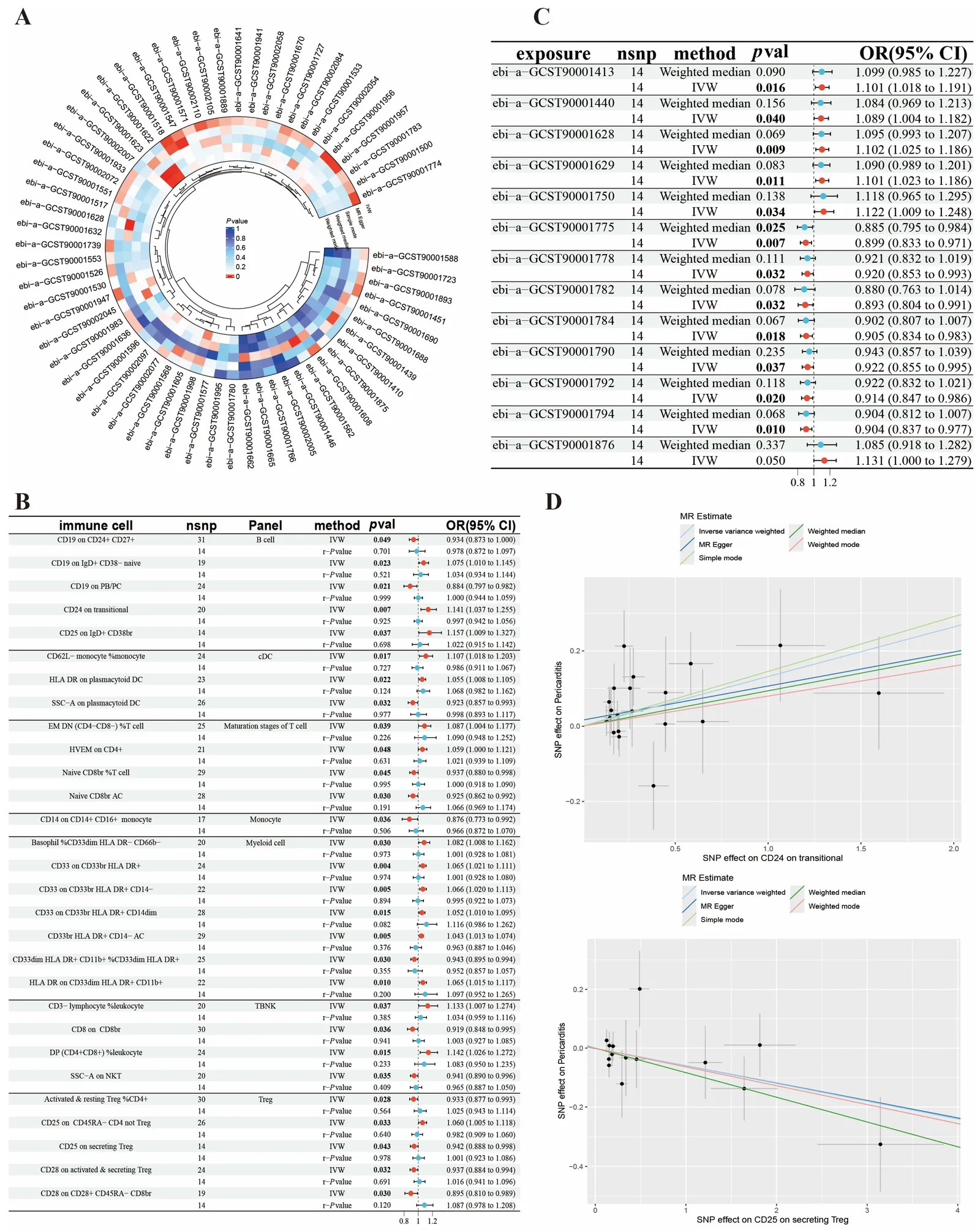
Genetic associations between immune cells and pericarditis revealed by MR. (**A**) Circos plot summarizing the effects of all immune cell traits nominally associated with pericarditis (*p* < 0.05), as estimated by five MR methods. (**B**) Forest plot displaying the genetically predicted effects of the 29 significant immunophenotypes on pericarditis from the primary IVW analysis. The *p*-value from the corresponding reverse MR analysis (Preverse) is annotated for each trait. Red dots represent the forward MR IVW estimates, and blue dots represent the reverse MR IVW estimates. The vertical dashed line indicates the null line (OR = 1), and the lines on either side of the dots represent 95% confidence intervals. (**C**) Forest plot of the effects of genetic susceptibility to pericarditis on the 13 immunophenotypes identified in reverse MR analysis. Red dots represent IVW estimates, and blue dots represent weighted median estimates. The vertical dashed line indicates the null line (OR = 1). (**D**) Scatter plots of SNP effects for two representative immunophenotypes. Each point represents an instrumental SNP, and the slope of each line corresponds to the effect estimate for a different MR method. Grey lines represent the 95% confidence intervals for each SNP effect estimate on the exposure and outcome. The upper panel shows CD24 on transitional B cell (a risk factor), and the lower panel shows CD25 on secreting Treg (a protective factor). IVW, inverse variance weighted.

We first conducted MR analyses to assess the potential causal link between immune cells and pericarditis. Following rigorous instrument selection (clumping and removal of weak instruments) 18,621 SNPs (minimum F-statistic = 19.59) were included for the immunophenotypes, resulting in robust instruments. The main IVW analysis identified 29 immunophenotypes with significant genetically predicted associations with pericarditis. These candidate causal immunophenotypes covered diverse immunological lineages, such as B cells (e.g., CD19 on PB/PC; CD24 on transitional), dendritic cells (HLA DR on plasmacytoid DC), T cells (Naive CD8br %T cell), myeloid cells (CD33 on CD33br HLA DR+), TBNK cells (CD8 on CD8br; CD3− lymphocyte %leukocyte), and regulatory T cells (CD25 on secreting Treg). After Benjamini–Hochberg FDR correction, all 29 immunophenotypes remained significantly associated with pericarditis (all FDR < 0.05) (Table S4). Among the phenotypes identified, 20 were risk factors for pericarditis, and 9 were protective factors. The robustness of these associations was supported by sensitivity analyses: (1) effect estimates were consistent in direction across all five MR methods; (2) for phenotypes with potential pleiotropy, the weighted median estimate yielded similar effect sizes; and (3) leave-one-out analysis confirmed that no association was driven by a single influential SNP. To sum up, this study provides robust genetic evidence for associations between multiple immunophenotypes and pericarditis (Table 1, Figure 2).

To assess the potential causal effects of pericarditis on the immune landscape and to rigorously evaluate reverse causality, two complementary reverse MR analyses were performed. First, a comprehensive scan using pericarditis as the exposure and all 731 immunophenotypes as outcomes identified 13 immunophenotypes that were significantly associated with genetic susceptibility to pericarditis (*p* < 0.05). After FDR correction, these 13 reverse associations remained significant (all FDR < 0.05). These affected phenotypes included IgD− CD24− %B cell and IgD− CD24− %lymphocyte in the B-cell category, HLA DR+ CD8br %T cell and HLA DR+ CD8br %lymphocyte in the TBNK category, as well as CD25 expression levels on several B-cell subsets (e.g., CD25 on B cell, CD25 on memory B cell). Notably, there was a complete lack of overlap between these 13 immunophenotypes, which are consequences of the disease, and the 29 immunophenotypes identified as genetically predicted risk factors (Supplementary Tables S2 and S3). Second, we specifically tested for reverse causality affecting the 29 candidate causal immunophenotypes to ensure their validity as exposures for mediation analysis. No significant effects of pericarditis on these phenotypes were observed (*p* > 0.05). Together, these analyses delineate a clear bidirectional genetic architecture: one set of immune cells are genetically predicted risk factors for pericarditis, while a distinct set are consequences of the disease state. Furthermore, these analyses reduce concern that reverse causality biased the primary findings or subsequent mechanistic explorations. No significant horizontal pleiotropy was observed for these associations. In the reverse MR analysis restricted to the 29 candidate immune phenotypes, heterogeneity was absent for the majority of traits; only CD4+CD8+ T cell %leukocyte showed evidence of heterogeneity (Cochran’s Q *p* = 0.009). This phenotype was not part of the main mediation pathway. In these reverse MR analyses, 14 independent SNPs were used as instruments for pericarditis, with F-statistics ranging from 20.87 to 26.93.

### 3.2. Genetic Associations Between Pericarditis and Metabolites

**Figure 3:**
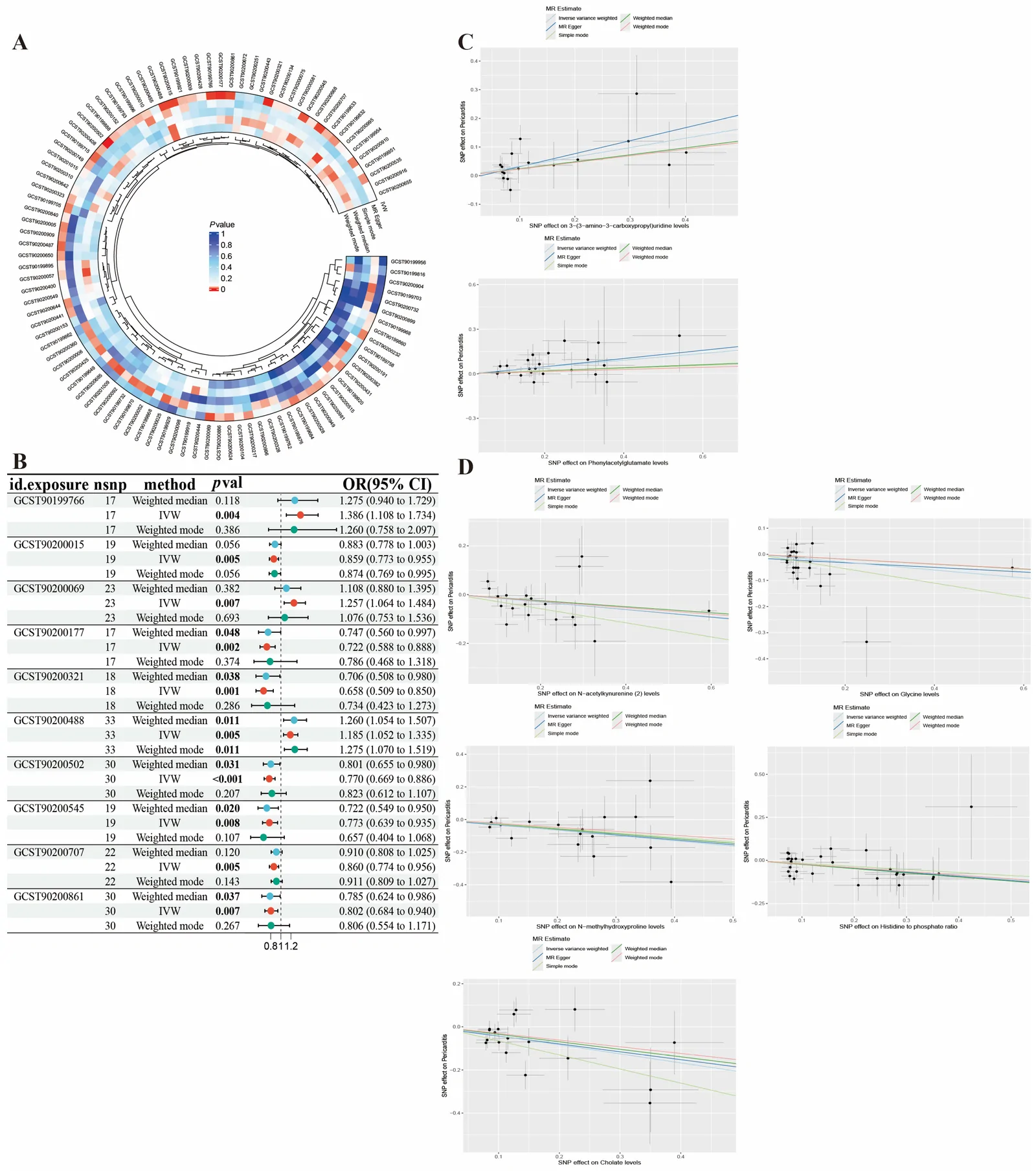
Genetic associations between pericarditis and plasma metabolites identified by MR. (**A**) Circos plot summarizing the 99 metabolites with at least one MR method showing nominal *p* < 0.05 in the metabolite-to-pericarditis analysis. (**B**) Forest plot showing the genetically predicted effects of the 10 significant metabolites on pericarditis estimated by IVW, weighted median, and weighted mode. (**C**) Scatter plots of SNP effects for the two known metabolite risk factors (acp^3^U and Phenylacetylglutamate). (**D**) Scatter plots of SNP effects for the five known metabolite protective factors (NAK, N-methylhydroxyproline, Cholate, Glycine, and the Histidine to phosphate ratio).

MR analyses were next performed to assess the potential causal relationship between plasma metabolites and pericarditis. Following instrument selection, 34,843 SNPs (minimum F-statistic = 19.52) for plasma metabolites were included in the analysis. The IVW analysis identified 10 plasma metabolites with significant potential causal relationships to pericarditis, including 7 known and 3 unknown metabolites. After FDR correction, all 10 metabolites remained significantly associated with pericarditis (all FDR < 0.05) (Table S4). Of the known metabolites, two were potential risk factors for pericarditis: 3-(3-amino-3-carboxypropyl)uridine (acp^3^U) and Phenylacetylglutamate. The other five known metabolites were potential protective factors: N-acetylkynurenine (NAK), N-methylhydroxyproline, Cholate, Glycine, and the Histidine to phosphate ratio. MR-Egger intercept tests revealed no evidence of horizontal pleiotropy for these associations, and Cochran’s Q tests showed no substantial heterogeneity for the reported metabolite associations (Table 2, Figure 3).

### 3.3. Mediation MR Analysis

A two-step MR approach was utilized to identify plasma metabolites that potentially mediate the effects of the candidate causal immunophenotypes on pericarditis. In the first step, we assessed the effects of the 29 candidate immunophenotypes on the 10 significant metabolites. This revealed 7 significant genetically predicted associations linking 6 immunophenotypes to 5 plasma metabolites (Table 3). Notable findings from this step included positive effects of CD24 on transitional on acp^3^U levels and of CD8 on CD8br on Glycine levels, alongside a negative effect of CD14 on CD14+ CD16+ monocyte on NAK levels. We also observed that certain immunophenotypes could influence multiple metabolites. For example, CD25 on CD45RA-CD4 not Treg exerted opposing effects, positively affecting Glycine levels while negatively affecting Cholate levels.

**Figure 4:**
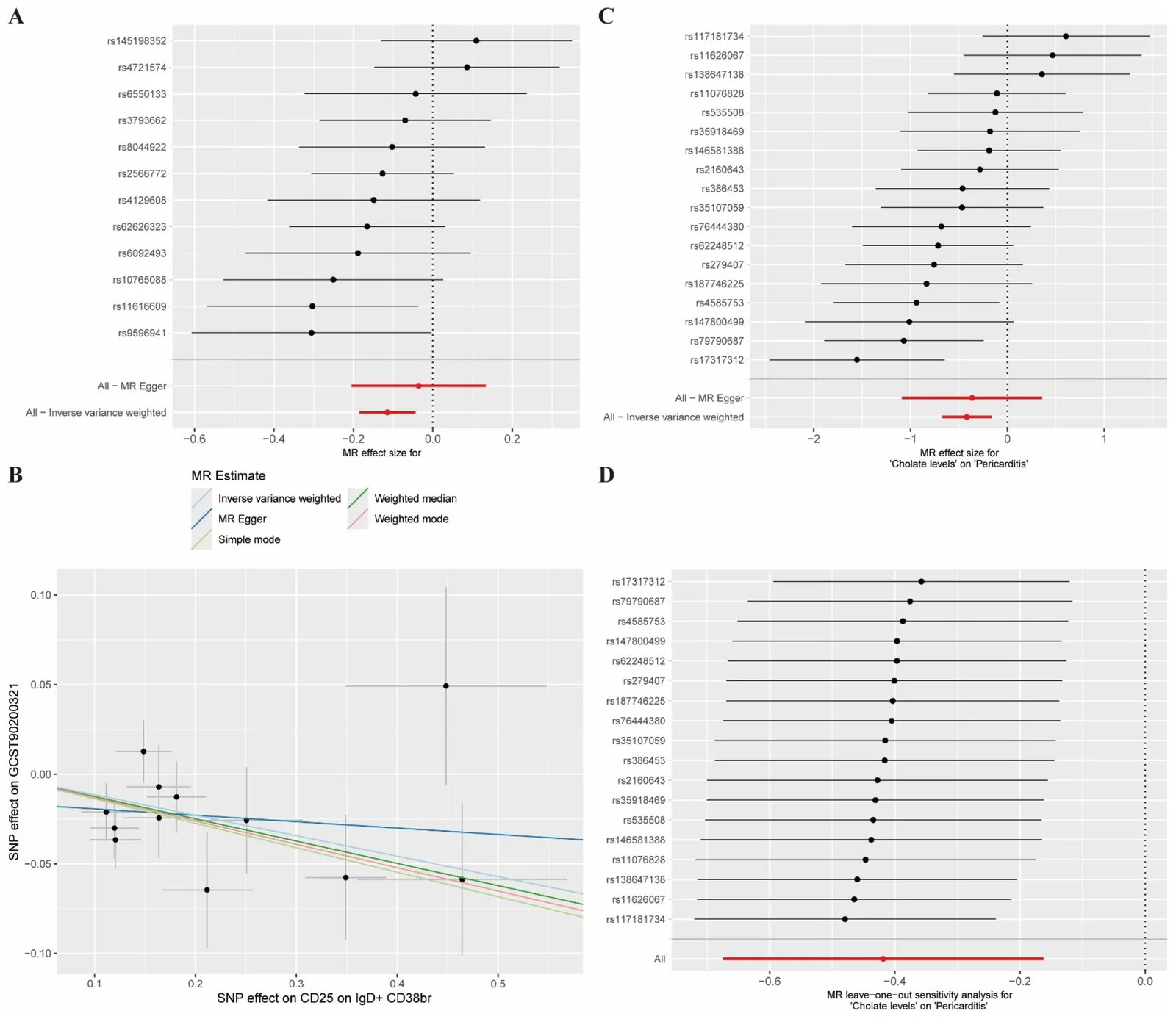
Genetic evidence for a potential pathway from CD25-positive B cells to pericarditis via cholate. (**A**) Forest plot and (**B**) scatter plot of the negative genetically predicted effect of CD25 on IgD+ CD38br on cholate levels (Step 1; path β_1_). (**C**) Forest plot of the protective genetically predicted effect of cholate on pericarditis (Step 2; path β_2_). (**D**) Leave-one-out sensitivity analysis for the cholate-pericarditis association (Step 2), demonstrating the result is not driven by a single influential SNP. Vertical dashed lines indicate the null line (β = 0). Grey lines represent 95% confidence intervals.

Among the seven significant immune cell–metabolite associations, the CD25 on IgD+ CD38br to cholate pathway was selected for formal mediation analysis because it showed the smallest IVW *p*-value, no significant heterogeneity or horizontal pleiotropy, and cholate itself was strongly protective against pericarditis. Based on the results of the first stage, we then analyzed the cholate concentration based on the CD25 on IgD+ CD38br pathway in subsequent analyses to further explore the mechanisms of mediation. This pathway showed a significant negative genetically predicted effect (IVW β = −0.114, SE = 0.036, *p* = 0.002), which is equivalent to effect size b1 of the mediation model. Corresponding sensitivity analyses indicated no evidence for horizontal pleiotropy (*p* = 0.340) or heterogeneity (*p* = 0.395). Finally, in order to finalize the mediation model, we conducted the second-step MR analysis in which cholate levels were used as exposure and pericarditis as outcome. Results showed that genetically predicted increased cholate levels were protective against pericarditis (IVW β = −0.419, SE = 0.131, *p* = 0.001), which is equivalent to effect size b2 from metabolite to disease. For the cholate-to-pericarditis association, MR-PRESSO global test showed no significant horizontal pleiotropy (*p* = 0.079), and no outlier SNP was detected (Table 4, Figure 4). Steiger directionality testing confirmed that the instrumental variables explained substantially more variance in the respective exposure than in the outcome for both the CD25 on IgD+ CD38br-to-pericarditis total effect and the cholate-to-pericarditis association.

Finally, we estimated the total effect of CD25 on IgD+ CD38br on pericarditis risk in the mediation model. The IVW method identified a significant positive total effect of this immunophenotype on pericarditis risk (β = 0.146, SE = 0.070, *p* = 0.037). The effect direction was consistent across supplementary MR methods, and sensitivity analyses indicated no significant horizontal pleiotropy (MR-Egger intercept *p* = 0.383) or heterogeneity (Cochran’s Q *p* = 0.886) (Table 4). Together with the estimates for path b1 (immunophenotype to cholate) and path b2 (cholate to pericarditis), this total effect estimate provided the complete genetic basis for quantifying the mediation effect.

### 3.4. Mediation Analysis

**Figure 5:**
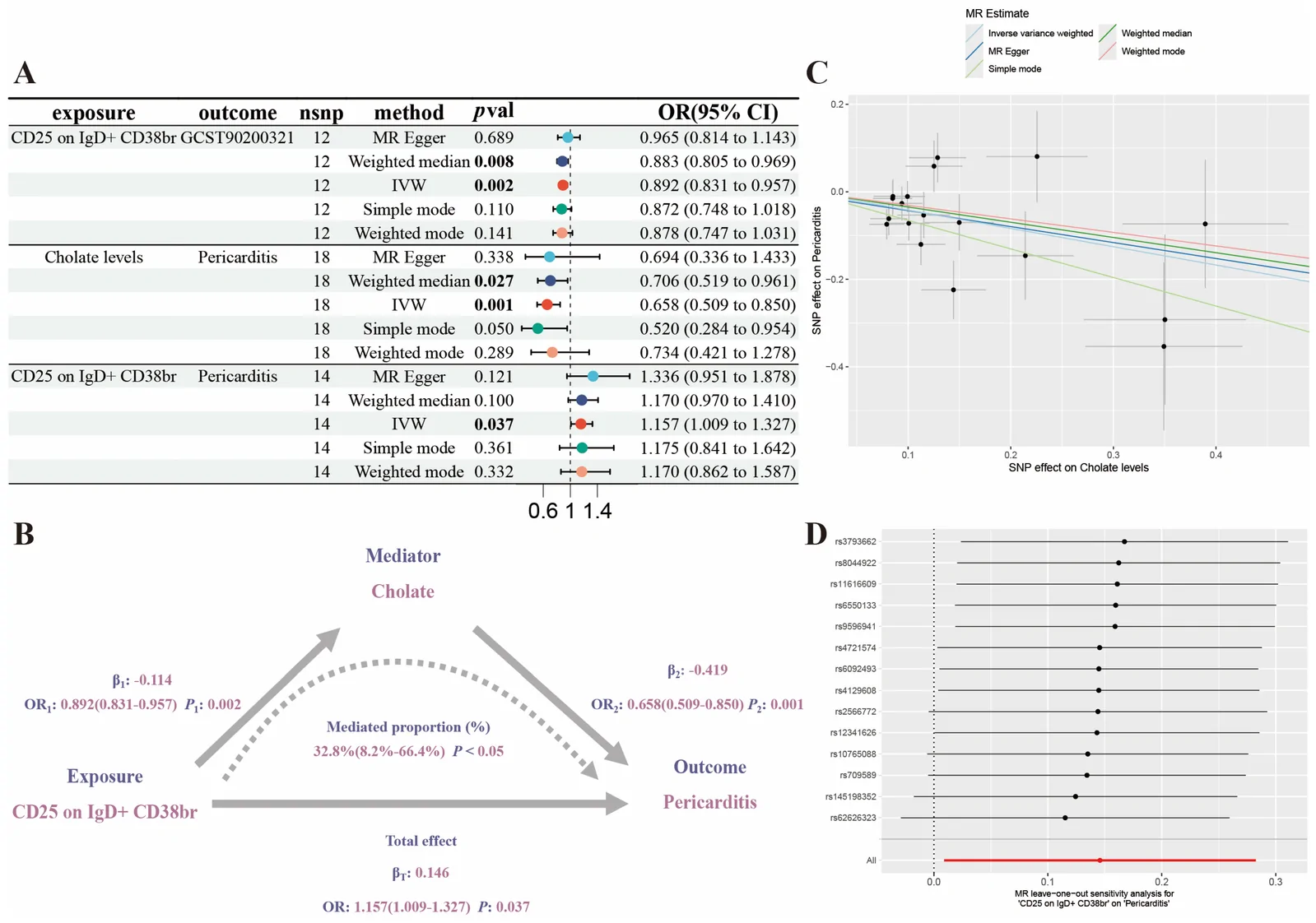
The mediating role of cholate in the potential pathway from CD25 on IgD+ CD38br B cells to pericarditis. (**A**) Forest plot displaying the effect estimates for the mediation model: the effect of the immunophenotype on cholate (β_1_), the effect of cholate on pericarditis (β_2_), and the total effect of the immunophenotype on pericarditis. (**B**) Schematic illustration of the mediation model presented in (**A**), showing path coefficients with standard errors and *p*-values from the IVW MR analysis. (**C**) Scatter plot of the genetic association for the cholate-pericarditis relationship (path β_2_). (**D**) Leave-one-out sensitivity analysis for the total effect of CD25 on IgD+ CD38br on pericarditis, confirming the robustness of the association. The pathway suggests that the immunophenotype increases pericarditis risk partly through reducing protective cholate levels.

Using the effect estimates derived from the two-step MR analyses, we formally quantified the extent of mediation in the potential pathway from immune cells to pericarditis. The results revealed a significant mediation pathway (Table 5). Specifically, the genetic effect of the B-cell phenotype CD25 on IgD+ CD38br on pericarditis was mediated through cholate. The mediation analysis revealed a significant indirect effect of 0.048 (95% CI: 0.012 to 0.097; *p* < 0.05), accounting for 32.8% of the total effect of CD25 on IgD+ CD38br on pericarditis risk. The direct effect was 0.098. This indicates that nearly one-third of this immunophenotype’s genetic effect on pericarditis is mediated by cholate. The negative estimates for path b1 (immunophenotype to cholate) and path b2 (cholate to pericarditis) are consistent with a model in which higher CD25 expression on these B cells increases pericarditis risk, partly by reducing circulating levels of the protective metabolite cholate (Figure 5).

## 4. Discussion

Using a comprehensive two-sample MR framework, we systematically examined potential causal pathways between immune cells and pericarditis and identified a possible mediating role of plasma metabolites. The main findings can be summarized as follows. First, forward MR analyses identified 29 immunophenotypes with genetically predicted associations with pericarditis, among which 20 were risk factors and 9 were protective factors. Second, reverse MR analyses suggested a bidirectional genetic architecture, in which genetic susceptibility to pericarditis was associated with changes in 13 immunophenotypes. Importantly, none of these 13 outcome-related immunophenotypes overlapped with the 29 candidate causal phenotypes, thereby reducing concern that reverse causation biased the major findings. Finally, mediation analysis identified a putative pathway from an immune cell phenotype through a plasma metabolite to pericarditis. Specifically, the B-cell phenotype CD25 on IgD+ CD38br was associated with increased pericarditis risk, partly through downregulation of the protective metabolite cholate. This mediation effect accounted for 32.8% of the total effect, supporting a potential role of cholate in this pathway.

These genetic findings led us to further consider the specific pathological roles of different immune cells in pericarditis. A detailed examination of the candidate causal immunophenotypes suggested that B cells, myeloid cells, and regulatory T cells are important immune lineages that may contribute to pericarditis risk. Importantly, our genetic data revealed functionally distinct B-cell subsets. CD19 on PB/PC and CD24 on transitional B cells were associated with increased pericarditis risk, whereas CD25 on IgD+ CD38br B cells also showed a genetically predicted risk-increasing effect, which was partly mediated by reduced cholate levels. These observations suggest that different B-cell functional states may exert distinct regulatory influences in pericarditis, although the exact mechanisms remain to be defined. While B cell and plasma cell infiltration in pericarditis has been reported in observational studies [22,23,24], these associations were prone to confounding and reverse causation. Our MR findings provide genetic evidence suggesting that specific B-cell states may contribute to pericarditis rather than merely reflecting disease consequences. In addition to B cells, our results support a potential protective role of regulatory T cells. Although animal studies have suggested that regulatory T cells may exert anti-inflammatory effects in the heart [25,26], direct human genetic evidence has been limited. Our findings suggest that the regulatory T-cell phenotype CD25 on secreting Treg is associated with reduced pericarditis risk, supporting its potential protective role. This interpretation is consistent with reports of immune checkpoint inhibitor–associated pericarditis, in which immune dysregulation may promote regulatory T-cell dysfunction, enhance autoimmune responses, and aggravate pericardial inflammation [27,28]. Taken together, these findings refine the immunological model of pericarditis and identify distinct immune cell subsets, particularly specific B-cell programs and regulatory T cells, as potential targets for future investigation.

Having identified genetically predicted effects of immune phenotypes on pericarditis risk, we then examined whether genetic susceptibility to pericarditis was associated with subsequent immune alterations. Reverse MR analyses showed that genetic susceptibility to pericarditis was associated with 13 immunophenotypes, none of which overlapped with the forward-identified candidate phenotypes. These 13 phenotypes may therefore represent a disease-related immune remodeling profile. A consistent pattern was observed, suggesting that the predominant immune changes occurred in the B-cell lineage. This remodeling was characterized by broad alterations in the B-cell compartment, including downregulation of CD25, a key activation marker, across multiple B-cell subsets, together with shifts in population frequencies, namely an increase in IgD− CD24− B cells and activated HLA DR+ CD8br cells. This potential bidirectional immune dysregulation suggests that an initial immune-mediated pericarditis insult may be followed by counter-regulatory immune changes that could contribute to disease persistence. The coexistence of broad B-cell alterations and T-cell activation may be relevant to the chronicity and recurrence of pericarditis. Recurrent and chronic pericarditis are thought to involve autoimmune or autoinflammatory mechanisms [29], supported by the detection of anti-heart autoantibodies [30] and the efficacy of IL-1 pathway blockade [31,32,33]. These observations suggest that a self-perpetuating loop involving autoantibodies, inflammatory cells, and cytokines may contribute to chronicity [34]. In this context, the observed B-cell alterations may not be merely an epiphenomenon but could contribute to chronicity, possibly through loss of regulatory B-cell subsets that help maintain immune tolerance [35]. Thus, together with previous clinical observations [36], our genetic findings provide evidence consistent with pericarditis susceptibility influencing these specific immune alterations, although the temporal and mechanistic relationships require further investigation. This disease-associated immune signature may offer a basis for understanding progression and could inform the development of circulating biomarkers.

To explore potential downstream effector mechanisms, we examined the plasma metabolome as a possible mediator of immune cell effects. Among the pathways identified by the systematic screen, the pathway from CD25 on IgD+ CD38br B cells to cholate and then to pericarditis was the most statistically robust. This B-cell phenotype was associated with increased disease risk and decreased levels of the protective metabolite cholate. In addition to its role in lipid digestion, cholate has been proposed to modulate immune responses [37,38,39]. Our data identify cholate as a genetically predicted protective factor in pericarditis. Several mechanisms may explain this observation. For example, related bile acids have been reported to promote macrophage polarization toward an anti-inflammatory M2 phenotype [40,41], to regulate innate immunity through receptors such as TLR2 [42], and to modulate systemic inflammatory responses and endothelial integrity [43]. Cholate may also be relevant to the proposed gut–heart axis, because bile acids are important host-derived metabolites that undergo modification by the gut microbiota [44,45]. Our human genetic data provide evidence consistent with a potential role of cholate as a circulating mediator in the broader hypothesis linking gut microbiota, bile acid metabolism, and cardiac inflammation. This study therefore identifies a putative immune–metabolic axis in which CD25 on IgD+ CD38br B cells may increase pericarditis risk, partly through reduced circulating cholate levels. The identification of this immune–metabolic axis may provide a basis for further investigation into the pathogenesis of pericarditis, particularly the interplay between specific immune cells, systemic metabolic regulation, and local pericardial inflammation. The observation that genetically predicted lower cholate levels were associated with increased pericarditis risk, together with reports that very high bile acid concentrations can be pro-inflammatory [46], suggests that bile acid homeostasis, rather than a single directional effect, may be important for cardiac immune balance.

This study has several limitations that should be considered. First, although the MR analyses provide genetic evidence consistent with a potential causal pathway, the molecular mechanisms linking CD25 on IgD+ CD38br B cells to cholate metabolism remain to be determined. Second, the discovery screening used relaxed instrument selection thresholds and nominal *p*-value thresholds. Although key findings remained significant after FDR correction and were supported by multiple sensitivity analyses, some of the screened associations may still represent false positives. Third, although Steiger filtering was performed for key pathway analyses, formal colocalization analysis was not conducted because suitable tissue-specific or protein quantitative trait locus datasets that could be unambiguously matched to the immune cell phenotypes and metabolites examined in this study were not available. Therefore, the possibility that the identified genetic associations may be influenced by residual linkage or pleiotropic effects cannot be completely excluded. In addition, although MR-PRESSO and MR-Egger intercept tests did not detect significant pleiotropy, the genetic instruments for cholate may still be influenced by hepatic, metabolic, dietary, or gut microbiota-related pathways, and residual pleiotropy cannot be completely excluded. Fourth, the pericarditis GWAS dataset includes a relatively small number of cases and does not allow stratification by specific etiologies. Our findings therefore reflect genetic susceptibility to pericarditis in the general population and may not be directly applicable to rare or secondary forms of the disease. Fifth, all analyses were based on European ancestry populations. Whether these findings can be generalized to other populations requires confirmation in independent, multi-ancestry studies. Finally, this study did not include experimental validation. The putative B cell–cholate–pericarditis axis should therefore be interpreted as a genetically supported hypothesis rather than as an established biological mechanism.

## 5. Conclusions

This study systematically explored the genetic relationships between immune cells and pericarditis and identified a putative cholate-mediated pathway involving CD25-positive B cells. These findings may improve our understanding of the immunopathological basis of pericarditis and may inform future research on immunometabolic targets for prevention and treatment.

## Figures and Tables

**Table 1:** MR estimates for the associations of immune cell phenotypes with pericarditis.

Exposure	Method	Nsnp	Beta	Se	*p*-Val	Pleiotropy	Heterogeneity
CD19 on CD24+ CD27+	IVW	31	−0.068	0.035	0.049	0.644	0.968
CD33 on CD33br HLA DR+	IVW	24	0.063	0.022	0.004	0.534	0.818
CD25 on IgD+ CD38br	IVW	14	0.146	0.070	0.037	0.383	0.886
CD8 on CD8br	IVW	30	−0.085	0.041	0.036	0.664	0.053
CD14 on CD14+ CD16+ monocyte	IVW	17	−0.133	0.064	0.036	0.741	0.532
EM DN (CD4−CD8−) %T cell	IVW	25	0.084	0.041	0.039	0.583	0.711
SSC-A on NKT	IVW	20	−0.060	0.029	0.035	0.673	0.825
CD25 on CD45RA-CD4 not Treg	IVW	26	0.058	0.027	0.033	0.934	0.548
CD19 on IgD+ CD38− naive	IVW	19	0.073	0.032	0.023	0.462	0.697
CD62L-monocyte %monocyte	IVW	24	0.101	0.043	0.017	0.779	0.748
CD33 on CD33br HLA DR+ CD14−	IVW	22	0.064	0.022	0.005	0.525	0.731
Naive CD8br %T cell	IVW	29	−0.065	0.032	0.045	0.133	0.478
CD3− lymphocyte %leukocyte	IVW	20	0.125	0.060	0.037	0.793	0.042
CD28 on CD28+ CD45RA-CD8br	IVW	19	−0.110	0.051	0.030	0.812	0.482
CD33dim HLA DR+ CD11b+ %CD33dim HLA DR+	IVW	25	−0.058	0.027	0.030	0.404	0.864
HLA DR on plasmacytoid DC	IVW	23	0.054	0.023	0.022	0.936	0.792
CD19 on PB/PC	IVW	24	−0.123	0.053	0.021	0.572	0.965
Basophil %CD33dim HLA DR-CD66b-	IVW	20	0.079	0.036	0.030	0.761	0.902
CD24 on transitional	IVW	20	0.132	0.049	0.007	0.493	0.617
HVEM on CD4+	IVW	21	0.057	0.029	0.048	0.244	0.528
CD25 on secreting Treg	IVW	14	−0.060	0.030	0.043	0.952	0.751
CD33br HLA DR+ CD14-AC	IVW	29	0.042	0.015	0.005	0.375	0.568
SSC-A on plasmacytoid DC	IVW	26	−0.080	0.038	0.032	0.444	0.455
Activated & resting Treg %CD4+	IVW	30	−0.069	0.032	0.028	0.563	0.904
HLA DR on CD33dim HLA DR+ CD11b+	IVW	22	0.063	0.024	0.010	0.923	0.340
CD33 on CD33br HLA DR+ CD14dim	IVW	28	0.050	0.021	0.015	0.214	0.391
CD28 on activated & secreting Treg	IVW	24	−0.065	0.030	0.032	0.424	0.383
DP (CD4+CD8+) %leukocyte	IVW	24	0.133	0.055	0.015	0.634	0.552
Naive CD8br AC	IVW	28	−0.078	0.036	0.030	0.698	0.936

Notes: MR estimates were obtained using the IVW method. Effect estimates are expressed per standard deviation increase in the respective immune cell phenotype. Pleiotropy *p*-values were derived from MR-Egger intercept tests, and heterogeneity *p*-values were derived from Cochran’s Q tests. CD33-related phenotypes represent distinct immune cell subsets defined by the indicated marker combinations; they correspond to different GWAS identifiers. Nsnp, number of single-nucleotide polymorphisms used as instrumental variables; Beta, effect estimate; Se, standard error.

**Table 2:** MR estimates for the associations of plasma metabolites with pericarditis.

Exposure	Method	Nsnp	Beta	Se	*p*-Val	Pleiotropy	Heterogeneity
3-(3-amino-3-carboxypropyl)uridine levels	IVW	17	0.327	0.114	0.004	0.585	0.648
N-acetylkynurenine (2) levels	IVW	19	−0.152	0.054	0.005	0.942	0.355
Phenylacetylglutamate levels	IVW	23	0.229	0.085	0.007	0.803	0.967
N-methylhydroxyproline levels	IVW	17	−0.325	0.105	0.002	0.902	0.562
Cholate levels	IVW	18	−0.419	0.131	0.001	0.878	0.063
X-12117 levels	IVW	33	0.170	0.061	0.005	0.490	0.972
X-12707 levels	IVW	30	−0.261	0.072	0.0003	0.143	0.907
X-17438 levels	IVW	19	−0.257	0.097	0.008	0.926	0.525
Glycine levels	IVW	22	−0.151	0.054	0.005	0.267	0.402
Histidine to phosphate ratio	IVW	30	−0.220	0.081	0.007	0.903	0.425

Notes: MR estimates were obtained using IVW. Pleiotropy *p*-values were derived from MR-Egger intercept tests, and heterogeneity *p*-values were derived from Cochran’s Q tests. Nsnp, number of single-nucleotide polymorphisms used as instrumental variables; Beta, effect estimate; Se, standard error.

**Table 3:** MR estimates for the effect of immune cells on metabolite levels.

Exposure	Outcome	Method	Nsnp	Beta	Se	*p*-Val	Pleiotropy	Heterogeneity
CD25 on IgD+ CD38br	Cholate levels	IVW	12	−0.114	0.036	0.002	0.340	0.395
CD8 on CD8br	Glycine levels	IVW	28	0.031	0.015	0.039	0.215	0.638
CD14 on CD14+ CD16+ monocyte	N-acetylkynurenine (2) levels	IVW	16	−0.075	0.033	0.026	0.969	0.647
SSC-A on NKT	X-12117 levels	IVW	20	0.030	0.015	0.044	0.960	0.228
CD25 on CD45RA- CD4 not Treg	Cholate levels	IVW	26	−0.026	0.013	0.040	0.881	0.413
CD25 on CD45RA- CD4 not Treg	Glycine levels	IVW	26	0.027	0.012	0.024	0.372	0.426
CD24 on transitional	3-(3-amino-3-carboxypropyl) uridine levels	IVW	20	0.069	0.028	0.013	0.985	0.023

Notes: MR estimates were obtained using IVW. Pleiotropy *p*-values were derived from MR-Egger intercept tests, and heterogeneity *p*-values were derived from Cochran’s Q tests. Nsnp, number of single-nucleotide polymorphisms used as instrumental variables; Beta, effect estimate; Se, standard error.

**Table 4:** Underlying MR Estimates for the Potential Pathway from CD25 on IgD+ CD38br to Pericarditis.

Exposure	Outcome	Method	Nsnp	Beta	Se	*p*-Val	Pleiotropy	Heterogeneity
CD25 on IgD+ CD38br	Cholate levels	IVW	12	−0.114	0.036	0.002	0.340	0.395
Cholate levels	Pericarditis	IVW	18	−0.419	0.131	0.001	0.878	0.063
CD25 on IgD+ CD38br	Pericarditis	IVW	14	0.146	0.07	0.037	0.383	0.886

Notes: MR estimates were obtained using IVW. Pleiotropy *p*-values were derived from MR-Egger intercept tests, and heterogeneity *p*-values were derived from Cochran’s Q tests. MR-PRESSO and Steiger directionality results for the key pathway are described in the main text. Nsnp, number of single-nucleotide polymorphisms used as instrumental variables; Beta, effect estimate; Se, standard error.

**Table 5:** Results of the Mediation Analysis for the Effect of Immune Cells on Pericarditis via Plasma Metabolites.

Immune Cell	Metabolite	Outcome	Mediated Effect	Mediated Proportion	*p*-Value
CD25 on IgD+ CD38br	Cholate levels	Pericarditis	0.048 (0.012, 0.097)	32.8% (8.2%, 66.4%)	*p* < 0.05

## Data Availability

The GWAS summary statistics used in this study are publicly available from the GWAS Catalog. The pericarditis outcome data are available under accession ID ebi-a-GCST90018896. Immune cell phenotype data correspond to accession IDs GCST90001391–GCST90002121, and plasma metabolite data correspond to accession IDs GCST90199621–GCST90201020.

## Supplementary Materials

The following supporting information can be downloaded at: https://www.mdpi.com/article/10.3390/biomedicines14092063/s1, Supplementary Table S1. STROBE-MR checklist of recommended items to address in reports of Mendelian randomization studies; Supplementary Table S2. Results of the comprehensive reverse MR analysis of pericarditis on 731 immune cell phenotypes; Supplementary Table S3. Results of reverse Mendelian randomization analysis assessing the effect of genetic liability to pericarditis on the 29 candidate immune cell traits; Supplementary Table S4. Benjamini–Hochberg FDR-corrected associations for the main immune cell and metabolite findings, and Steiger directionality testing for the key pathway.
