# Supplementary material for: Genetic Evidence for a Putative B Cell–Cholate Immunometabolic Axis in Pericarditis: A Mendelian Randomization Study
Source: Biomedicines. 2026 Sep 14;14(9):2063. doi: 10.3390/biomedicines14092063 (PMC13604588; doi:10.3390/biomedicines14092063)
Supplement: Supplementary file 1 [file biomedicines-14-02063-s001.zip › Supplementary Table S2.pdf]

Supplementary Table S2. Results of the comprehensive reverse MR analysis of pericarditis on 731 immune cell phenotypes.

**Outcome**	**Method**	**Nsnp**	**Beta**	**Se**	***P*-val**	**Pleiotropy**	**Heterogeneity**	**FDR**
IgD- CD24- %B cell	IVW	14	0.096	0.040	0.016	0.675	0.415	0.037
IgD- CD24- %lymphocyte	IVW	14	0.086	0.042	0.040	0.846	0.352	0.043
HLA DR+ CD8br %T cell	IVW	14	0.097	0.037	0.009	0.871	0.761	0.035
HLA DR+ CD8br %lymphocyte	IVW	14	0.096	0.038	0.011	0.783	0.789	0.035
CD20 on IgD+ CD38- unsw mem	IVW	14	0.115	0.054	0.034	0.995	0.965	0.043
CD25 on B cell	IVW	14	-0.106	0.039	0.007	0.881	0.881	0.035
CD25 on IgD+ CD24+	IVW	14	-0.083	0.039	0.032	0.614	0.976	0.043
CD25 on IgD+ CD38- unsw mem	IVW	14	-0.114	0.053	0.032	0.926	0.641	0.043
CD25 on IgD+ CD38dim	IVW	14	-0.099	0.042	0.018	0.974	0.330	0.037
CD25 on memory B cell	IVW	14	-0.081	0.039	0.037	0.672	0.995	0.043
CD25 on unsw mem	IVW	14	-0.090	0.039	0.020	0.469	0.962	0.037
CD25 on IgD+	IVW	14	-0.101	0.039	0.010	0.826	0.664	0.035
HVEM on CM CD4+	IVW	14	0.123	0.063	0.050	0.788	0.871	0.050

Notes: MR estimates were obtained using IVW. Pleiotropy *P*-values were derived from MR-Egger intercept tests, and heterogeneity *P*-values were derived from Cochran's Q tests. FDR values were calculated using the Benjamini–Hochberg method. Nsnp, number of single-nucleotide polymorphisms used as instrumental variables; Beta, effect estimate; Se, standard error.
