# Supplementary material for: Genetic Evidence for a Putative B Cell–Cholate Immunometabolic Axis in Pericarditis: A Mendelian Randomization Study
Source: Biomedicines. 2026 Sep 14;14(9):2063. doi: 10.3390/biomedicines14092063 (PMC13604588; doi:10.3390/biomedicines14092063)
Supplement: Supplementary file 1 [file biomedicines-14-02063-s001.zip › Supplementary Table S3.pdf]

Supplementary Table S3. Results of reverse Mendelian randomization analysis assessing the effect of genetic liability to pericarditis on the 29 candidate immune cell traits.

Outcome	ID	Method	Nsnp	Beta	SE	*P*-value	Pleiotropy	Heterogeneity
CD62L- monocyte %monocyte	ebi-a-GCST90001451	IVW	14	0.025	0.043	0.564	0.777	0.994
Activated & resting Treg %CD4+	ebi-a-GCST90001500	IVW	14	0.001	0.039	0.973	0.500	0.826
CD33br HLA DR+ CD14- AC	ebi-a-GCST90001518	IVW	14	-0.035	0.052	0.506	0.861	0.466
CD33dim HLA DR+ CD11b+ %CD33dim HLA DR+	ebi-a-GCST90001526	IVW	14	-0.022	0.059	0.701	0.428	0.256
Basophil %CD33dim HLA DR- CD66b-	ebi-a-GCST90001533	IVW	14	0.033	0.052	0.521	0.225	0.870
Naive CD8br AC	ebi-a-GCST90001551	IVW	14	0.000	0.029	0.999	0.055	0.265
Naive CD8br %T cell	ebi-a-GCST90001553	IVW	14	-0.003	0.029	0.925	0.330	0.270
EM DN (CD4-CD8-) %T cell	ebi-a-GCST90001571	IVW	14	-0.018	0.039	0.640	0.354	0.401
DP (CD4+CD8+) %leukocyte	ebi-a-GCST90001608	IVW	14	0.022	0.057	0.698	0.814	0.009
CD3- lymphocyte %leukocyte	ebi-a-GCST90001641	IVW	14	0.001	0.041	0.978	0.835	0.360
CD19 on CD24+ CD27+	ebi-a-GCST90001723	IVW	14	0.016	0.039	0.691	0.462	0.954
CD19 on IgD+ CD38- naive	ebi-a-GCST90001727	IVW	14	0.084	0.054	0.120	0.437	0.694
CD19 on PB/PC	ebi-a-GCST90001739	IVW	14	0.034	0.039	0.385	0.245	0.645
CD24 on transitional	ebi-a-GCST90001774	IVW	14	0.001	0.039	0.974	0.200	0.813
CD25 on IgD+ CD38br	ebi-a-GCST90001783	IVW	14	-0.005	0.039	0.894	0.476	0.948
HVEM on CD4+	ebi-a-GCST90001875	IVW	14	0.109	0.063	0.082	0.550	0.806
CD28 on activated & secreting Treg	ebi-a-GCST90001889	IVW	14	-0.037	0.042	0.376	0.246	0.750
CD28 on CD28+ CD45RA- CD8br	ebi-a-GCST90001893	IVW	14	-0.050	0.054	0.355	0.404	0.105
CD25 on CD45RA- CD4 not Treg	ebi-a-GCST90001933	IVW	14	-0.014	0.040	0.727	0.902	0.838
CD25 on secreting Treg	ebi-a-GCST90001941	IVW	14	0.003	0.040	0.941	0.484	0.920
CD33 on CD33br HLA DR+ CD14dim	ebi-a-GCST90001947	IVW	14	0.080	0.067	0.233	0.716	0.202
CD33 on CD33br HLA DR+	ebi-a-GCST90001956	IVW	14	0.086	0.071	0.226	0.800	0.120
CD33 on CD33br HLA DR+ CD14-	ebi-a-GCST90001957	IVW	14	0.093	0.073	0.200	0.798	0.096
CD14 on CD14+ CD16+ monocyte	ebi-a-GCST90001983	IVW	14	0.066	0.043	0.124	0.055	0.253
CD8 on CD8br	ebi-a-GCST90002058	IVW	14	0.020	0.042	0.631	0.315	0.440
SSC-A on plasmacytoid DC	ebi-a-GCST90002072	IVW	14	0.000	0.044	0.995	0.057	0.668
SSC-A on NKT	ebi-a-GCST90002084	IVW	14	0.064	0.049	0.191	0.970	0.192
HLA DR on plasmacytoid DC	ebi-a-GCST90002105	IVW	14	-0.036	0.043	0.409	0.678	0.689
HLA DR on CD33dim HLA DR+ CD11b+	ebi-a-GCST90002110	IVW	14	-0.002	0.057	0.977	0.287	0.624

Notes: MR estimates were obtained using IVW. Pleiotropy *P*-values were derived from MR-Egger intercept tests, and heterogeneity *P*-values were derived from Cochran's Q tests. Nsnp, number of single-nucleotide polymorphisms used as instrumental variables; Beta, effect estimate; SE, standard error.
