# Supplementary material for: Genetic Evidence for a Putative B Cell–Cholate Immunometabolic Axis in Pericarditis: A Mendelian Randomization Study
Source: Biomedicines. 2026 Sep 14;14(9):2063. doi: 10.3390/biomedicines14092063 (PMC13604588; doi:10.3390/biomedicines14092063)
Supplement: Supplementary file 1 [file biomedicines-14-02063-s001.zip › Supplementary Table S4.pdf]

Supplementary Table S4. Benjamini–Hochberg FDR-corrected associations for the main immune cell and metabolite findings, and Steiger directionality testing for the key pathway.

Part A. FDR-corrected associations for the 29 immune cell phenotypes

Exposure	IVW P	FDR
CD19 on CD24+ CD27+	0.049	0.049
CD33 on CD33br HLA DR+	0.004	0.045
CD25 on IgD+ CD38br	0.037	0.045
CD8 on CD8br	0.036	0.045
CD14 on CD14+ CD16+ monocyte	0.036	0.045
EM DN (CD4-CD8-) %T cell	0.039	0.045
SSC-A on NKT	0.035	0.045
CD25 on CD45RA- CD4 not Treg	0.033	0.045
CD19 on IgD+ CD38- naive	0.023	0.045
CD62L- monocyte %monocyte	0.017	0.045
CD33 on CD33br HLA DR+ CD14-	0.005	0.045
Naive CD8br %T cell	0.045	0.048
CD3- lymphocyte %leukocyte	0.037	0.045
CD28 on CD28+ CD45RA- CD8br	0.030	0.045
CD33dim HLA DR+ CD11b+ %CD33dim HLA DR+	0.030	0.045
HLA DR on plasmacytoid DC	0.022	0.045
CD19 on PB/PC	0.021	0.045
Basophil %CD33dim HLA DR- CD66b-	0.030	0.045
CD24 on transitional	0.007	0.045
HVEM on CD4+	0.048	0.049
CD25 on secreting Treg	0.043	0.048
CD33br HLA DR+ CD14- AC	0.005	0.045
SSC-A on plasmacytoid DC	0.032	0.045
Activated & resting Treg %CD4+	0.028	0.045

HLA DR on CD33dim HLA DR+ CD11b+	0.010	0.045
CD33 on CD33br HLA DR+ CD14dim	0.015	0.045
CD28 on activated & secreting Treg	0.032	0.045
DP (CD4+CD8+) %leukocyte	0.015	0.045
Naive CD8br AC	0.030	0.045

Part B. FDR-corrected associations for the 10 plasma metabolites

Exposure	IVW *P*	FDR
3-(3-amino-3-carboxypropyl)uridine levels	0.004	0.007
N-acetylkynurenine (2) levels	0.005	0.007
Phenylacetylglutamate levels	0.007	0.008
N-methylhydroxyproline levels	0.002	0.007
Cholate levels	0.001	0.007
X-12117 levels	0.005	0.007
X-12707 levels	0.0003	0.003
X-17438 levels	0.008	0.008
Glycine levels	0.005	0.007
Histidine to phosphate ratio	0.007	0.008

Part C. Steiger directionality testing for the key pathway

Pathway	SNP-exposure R^2	SNP-outcome R^2	Correct causal direction	Steiger *P*
CD25 on IgD+ CD38br → Pericarditis	0.128	4.0×10^{-5}	Yes	1.35×10^{-108}

Notes: IVW, inverse variance weighted; FDR, false discovery rate. FDR values were calculated using the Benjamini–Hochberg method. Steiger directionality testing confirmed that the instruments explained substantially more variance in the exposure than in the outcome for the key total-effect pathway. For the cholate-to-pericarditis relationship, Steiger testing also supported the assumed causal direction.
